# Ooplasmic transfer in human oocytes: efficacy and concerns in assisted reproduction

**DOI:** 10.1186/s12958-017-0292-z

**Published:** 2017-10-02

**Authors:** Sara Darbandi, Mahsa Darbandi, Hamid Reza Khorram Khorshid, Mohammad Reza Sadeghi, Ashok Agarwal, Pallav Sengupta, Safaa Al-Hasani, Mohammad Mehdi Akhondi

**Affiliations:** 1 Reproductive Biotechnology Research Center, Avicenna Research Institute, ACECR, Tehran, Iran; 2 Genetics Research Center, University of Social Welfare and Rehabilitation Sciences, Tehran, Iran; 3 Center for Reproductive Medicine, Glickman Urological and Kidney Institute, Cleveland Clinic, Cleveland, OH USA; 4 Physiology Unit Faculty of Medicine Lincoln University College, Petaling Jaya, Malaysia; 5 Reproductive Medicine Unit, University of Schleswig-Holstein, Luebeck, Germany

**Keywords:** Ooplasmic transfer, Mitochondria, Apoptosis, Genetic modifications, Epigenetic modifications

## Abstract

**Background:**

Ooplasmic transfer (OT) technique or cytoplasmic transfer is an emerging technique with relative success, having a significant status in assisted reproduction. This technique had effectively paved the way to about 30 healthy births worldwide. Though OT has long been invented, proper evaluation of the efficacy and risks associated with this critical technique has not been explored properly until today. This review thereby put emphasis upon the applications, efficacy and adverse effects of OT techniques in human.

**Main body:**

Available reports published between January 1982 and August 2017 has been reviewed and the impact of OT on assisted reproduction was evaluated. The results consisted of an update on the efficacy and concerns of OT, the debate on mitochondrial heteroplasmy, apoptosis, and risk of genetic and epigenetic alteration.

**Short conclusion:**

The application of OT technique in humans demands more clarity and further development of this technique may successfully prove its utility as an effective treatment for oocyte incompetence.

## Capsule

This review study provided the efficacy and concerns regarding ooplasmic transfer (OT), the debate on mitochondrial heteroplasmy, apoptosis, and risk of genetic and epigenetic alterations.

## Background

The role of the ooplasm in oocyte maturation and activation is well known. Meiotic division from germinal vesicle (GV, 4 N) stage to second meiotic metaphase (MII, 2 N), fertilization and the embryonic genome activation are strictly controlled by ooplasmic regulators following maturation of nucleus and ooplasm [1]. Theoretically, ooplasmic transfer (OT), a technique that renders a poor quality oocyte by efficient transfer of essential cellular components, may be referred to as a partial ooplasmic transfer including messenger RNAs (mRNAs), proteins, energy-producing components, mitochondria, and several other important cellular organelles and innumerable undetected factors from healthy oocytes to the insufficient one. By the mentioned mechanism, the technique focuses on improving normal growth, viability as well as the overall quality of an earlier unhealthy oocyte so that the qualities required to successfully participate in formation of a healthy zygote are sufficient [1–5].

Mitochondria are maternally inherited organelles in ooplasm with their own genomes that provide adenosine triphosphate (ATP) within the cells via the oxidative phosphorylation (OXPHOS) pathway [6, 7]. Oocytes, on an average, have 100,000 mitochondria containing a single copy of mitochondrial DNA (mtDNA) [8, 9]. In mammals, mtDNA encodes 13 structural proteins, which are essential for high-level energy production in the cell [6]. Therefore, in some types of cells (e.g., immature oocytes and cleaving preimplantation embryos), mitochondrial activity directly affects viability [1, 7, 10]. The specific cell cycle factors in the donor ooplasm could improve the nuclear and ooplasmic maturation of the recipient oocytes according to the cell cycle phase [3, 4, 11, 12]. To meet these objectives, Muggleton-Harris in 1982 first attempted OT in mice where cytoplasm had been transferred from non-blocking to blocking embryo development [13]. Following the initiation, several experiments have been performed in assisted reproduction using animal or human oocytes aiming to enhance oocyte quality. But, still the detailed genetic mechanisms involved in OT which actually inculcate completeness in a disabled oocyte are blurred. While it is obvious that adopting an OT technique can practically establish normal growth and return viability to the embryos, this review explained the appropriate practical OT techniques used for human oocytes, and its positive and negative aspects in assisted reproduction.

### Ooplasmic transfer techniques

Since the first report of Muggleton-Harris in 1982 regarding the efficacy of OT, many reports had emerged covering such techniques in animal and human models. During the past 30 years, a variety of studies have been performed to overcome ooplasmic deficiencies and abnormalities in oocyte or embryo manipulation at the subcellular level [4, 13, 14]. The ability to improve the oocyte capacity through the transfer of donor ooplasmic components was first demonstrated in animals [13, 15]. In 1997, the human pregnancy was announced by Cohen et al. following the transfer of donor ooplasm into the oocytes of a patient [1]. After that, this method had been successfully used in patients with poor embryo development and recurrent implantation failure and the outcomes culminated in pregnancy and birth [1, 16–22]. Synchronous and asynchronous transfers are two types of OT techniques [15]. In synchronous transfer, the ooplasm of the donor replaces that of a recipient, both of which are at the same developmental stage (from fresh GV to aged GV or from young MII to post-mature MII) [15], while in asynchronous transfer, the replacement of ooplasm was done from one developmental staged oocyte to an oocyte lying at a different stage of development (from MII to prophase I (MI) [15]. Although studies have often been carried out on synchronous transfer, but the embryonic development potential of all the studies has been reported in Tables 1 and 2. For better interactions between the ooplasm and the nucleus, about 5–15% of ooplasm has been transferred and oocytes with twice of the volume had no increase in reprogramming potential [1, 4, 23]. According to previous studies, the cryopreserved human oocytes or three-pronuclei (3-PN) embryos can be used for donor and recipient synchronization [18, 19]. This technique is performed either by electrofusion of the ooplasm and then intra cytoplasmic sperm injection (ICSI) of the recipient oocyte or by injecting the donor ooplasm with a single spermatozoon into the recipient oocyte using a standard ICSI needle [1, 16]. In the second protocol, at first, a spermatozoon was immobilized and placed in an ICSI needle, followed by removal of about 5–15% of the donor ooplasm (from the vegetal pole in MII) by suction with the spermatozoon marking the top of the needle. Finally, the donor ooplasm and spermatozoon were carefully injected into the recipient oocyte (near the animal pole in MII). The eggs were cultured to observe a second polar body and two pronuclei and then moved to growth medium [1, 16].

**Table 1:** **Comparison of embryonic development potential after MI and MII ooplasmic transfer (OT) in mammalian sample**

Supplier information			Survival information							Pregnancy information			Explanation
Species	D	R	OT (%)	Fertilized (%)	2-cell (%)	4-cell (%)	6-cell (%)	8-cell (%)	Blastocyst (%)	ET (%)	pregnancy	Birth	
Monkey [4]	MII	MI	42	23 (54.8%)	–	–	–	–	–	–	4 (9.5%)	3 (7.1%)	–
	Sham MI		43	26 (60.5%)	–	–	–	–	–	–	0	0	–
	Control MI		37	30 (81%)	–	–	–	–	–	–	0	0	–
	MII	MII	22	22 (100%)	–	–	–	–	–	–	4 (18%)	4 (18%)	–
	Sham MII		18	18 (100%)	–	–	–	–	–	–	3 (16.7%)	3 (16.7%)	–
	Control MII		24	24 (100%)	–	–	–	–	–	–	6 (25%)	4 (16%)	
Human [1]	MII	MII	14(100)	9 (64.3%)	8 (57.1%)	8 (57.1%)	8 (57.1%)	8 (57.1%)	4 (28.6%)	4 (28.6%)	1 (7.1%)	1 (7.1%)	amniocyte nDNA and mtDNA fingerprinting
Human [16]	MII	MII	22 fused	15 (68.2%)	–	–	–	10 (46.4%)	–	3 (13.6%)	0	0	amniocyte nDNA and mtDNA fingerprinting
	MII	MII	48	38 (79.17%)	–	–	–	8(16.7)	–	5 (10.4%)	3 (6.3%)	1 (2.1%)	amniocyte nDNA and mtDNA fingerprinting
	Control MII		17	9 (52.9%)	–	–	–	0	–	–	–	–	–
Human [18]	Cryo_MII	MII	37	26 (70.3%)	–	–	–	–	–	17 (45.9%)	2	2 (5%)	–
Human [19]	3PN	MII	62	44 (80%)	39 (63%)	–	39 (63%)	–	–	39 (63%)	4 (6.4%)	4	amniocentesis and karyotyping
Human [20]	MII	MII	26	–	–	–	–	–	–	–	–	2 (7.7%)	Confocal microscopy and mtDNA fingerprinting
Human [21]	MII	MII	8	6 (75%)	6 (75%)	5 (62.5%)	4 (50%)	2 (25%)	–	4 (50%)	2 (25%)	2 (25%)	–
	MII Control		4	3 (75%)	3 (75%)	1 (25%)	–	–	–	–	–	–	–

Donor: “D”. Recipient: “R”

**Table 2:** Comparison of embryonic development potential after PN, 1-cell and 2-cell ooplasmic transfer (OT) in mammalian sample

Supplier information			Survival information							Pregnancy information			Explanation
Species	D	R	OT (%)	Fertilized (%)	2-cell (%)	4-cell (%)	6-cell (%)	8-cell (%)	Blastocyst (%)	ET (%)	pregnancy	delivery	
Mouse [13]	F1,1-cell	MF1,1-cell	58 (55%)	106	46 (43.8%)	11 (10.4%)	–	9 (8.5%)	0				
	MF1,1-cell control			84	84 (95.5%)	39 (44.3%)	–	38 (43.1%)	35 (39.8%)				
Mouse [13]	F1,2-cell	MF1,1-cell	42 (58%)	73	42 (58%)	31 (43%)	–	30 (41.7%)	17 (23.6%)				
	MF1,1-cell control			92	92 (100%)	13 (14.1%)	–	1 (1.09%)	1 (1.09%)				
Mouse [13]	F1,2-cell	F1.1-cell	18 (75%)	24	18 (75%)	2 (0.03%)	–	9 (8.5%)	0				
	F1,1-cell control			73	73 (100%)	72 (98.6%)	–	68 (93.1%)	59 (80.8%)				
Mouse [14]	F1,2-cell,G1	F1.1-cell,G1	–	267	–	–	–	–	72 (27%)				
	F1,2-cell,G1	F1.1-cell,G2	–	664	–	–	–	–	73 (11%)				
	F1,2-cell,S	F1.1-cell,S	–	800	–	–	–	–	40 (5%)				
	F1,2-cell,G2	F1.1-cell,G2	–	111	–	–	–	–	50 (45%)				
	F1.1-cell,		–	315	–	–	–	–	221 (70%)				
	F1,2-cell,G1	MF1.1-cell,G1	–	29	–	–	–	–	12 (42%)				
	F1,2-cell,G1	MF1.1-cell,S	–	800	–	–	–	–	8 (0%)				
	F1,2-cell,G1	MF1.1-cell,G2	–	128	–	–	–	–	28 (22%)				
	F1,2-cell,S	MF1.1-cell,G2	–	1300	–	–	–	–	13 (0%)				
	F1,2-cell,G2	MF1.1-cell,G2	–	1067	–	–	–	–	32 (3%)				
	MF1.1-cell,		–	67,500	–	–	–	–	270 (0.4%)				
	F1,2-cell,G1	CD1.1-cell,G2	–	40	–	–	–	–	20 (50%)				
	F1,2-cell,S	CD1.1-cell,G2	–	2600	–	–	–	–	26 (0%)				
	F1,2-cell,G2	CD1.1-cell,G2	–	89	–	–	–	–	46 (52%)				
	CD1,2-cell,G2	CD1.1-cell,G2	–	2523	–	–	–	–	40 (1.5%)				
	CD1.1-cell,		–	3350	–	–	–	–	134 (4%)				
Mouse [15]	PN	PN		224	–	–	–	–	193 (86.2%)	106 (47.3%)	66 (29.5%)	24 (10.7%)	
	MII	PN		419	–	–	–	–	306 (73.03%)	134 (32%)	91 (21.7%)	71 (17%)	
	GV	PN		224	–	–	–	–	109 (48.7%)	40 (17.9%)	11 (4.9%)	5 (2.2%)	
	PN control			1114	–	–	–	–	1020 (91.6%)	246 (22.1%)	135 (12.1%)	99 (8.91%)	

### Pros and cons of ooplasmic transfer

Several reports have sprung up with the positive and negative aspects of OT indicating its efficacy and safety concerns, but lack of concrete documentation of these facts of OT in human oocytes inspired the researchers to review the entire available reports regarding OT published between 1982 to August 2017 in English language and to scrutinize the pros and cons of this technique in human embryos. Only the report of Wei-ren et al. (2006) has briefly mentioned the problems and prospects of OT, but after that report more than a decade has elapsed during which a number of research reports came up with new observations of the problems and prospects. Though there is no report elucidating positive correlation between any congenital abnormalities and the OT [10, 11], recently few reports have elucidated certain concerns regarding its use in assisted reproduction, like mitochondrial functioning in zygote, regulation of apoptosis, genetic and epigenetic modifications, survival of maternal transcripts, proteins and ions which are the factors effecting oocytes due to mixing of two entities [24–28].

#### Mitochondrial functions

Mitochondria have eccentric role in fertilization, cell division, development of embryo by balancing pro- and anti-apoptotic factors and also stabilising oxidative stress (OS). It has been proposed that in OT technique, genome of resulting offspring contains nuclear DNA (nDNA) from both of the biological parents and mtDNA from the biological mother as well as from the donor [27, 29]. Research revealed that although a brief mitochondrial heteroplasmy occurs in the newly fertilized oocyte (with respect to the entire sperm tail into the ooplasm), there remains a high degree of its homoplasmy throughout the body [30–32]. Mitochondrial function is also regulated by a complex combination of nuclear and mitochondrial genes and survival of mitochondrial genotypes relies on nuclear constitution as well [27, 29]. The introduction of foreign mtDNA via OT can introduce a conflict between the control mechanisms for nDNA, recipient mtDNA and donor mtDNA, leading to unpredictable outcomes which raised concerns regarding the safety of this technique [20, 27, 29, 33–37]. Spontaneous rearrangements of mtDNA include deletions, insertions and duplications which are responsible for some diseases [33, 37, 38]. In oocytes, when the percentage of mtDNA rearrangements increases, a significant reduction in the efficiency of OXPHOS occurs following reactive oxygen species (ROS) induced damages [33, 37]. In the zygote, ROS can induce lipid peroxidation and disruption in DNA, RNA and protein functions [33, 37, 39]. Though Krimsky (2014) expressed concerns regarding the sparse empirical knowledge regarding heteroplasmy in OT in human oocytes, it was shown in few studies that OT related mitochondrial disease transmission due to mtDNA mutation has a very low population frequency (1:8000) and therefore a theoretical risk of these diseases associated with OT seems unlikely [29, 35]. Only a few published animal studies have observed heteroplamy induced cardiovascular mal-alterations and neuropsychiatric defects in offsprings [40]. Ferreira et al. (2010) threw a beam on the fate of the donor’s mtDNA mentioning that owing to a reduced proportion of transmitted donor mtDNA compared with its input, it may be indicated that the introduced mtDNA was lost at any stage between foetal development and adulthood either by random drift or by selective replicative disadvantages of the transplanted mtDNA [41]. Bredenoord et al. (2008), in this regard, referred to the positive impact of OT on mitochondrial diseases. They mentioned, after clarifying some conceptual issues, that OT and nuclear transfer prevent mtDNA disorders [42].

Reports have also suggested that women carrying a history of IVF failure generate poor quality oocytes containing inefficient mitochondria, resulting in decreased energy production [43]. Healthy mitochondria are essential for apoptotic-signalling pathway and appropriate chromatid segregation during fertilization as well as subsequent mitotic division via respiratory processes and ATP production [44]. To focus upon the positive aspects of OT, it may thus be conceived that aged or defective mitochondria which may give rise to aberrant chromosomal segregation or developmental arrest, can become healthy with restored mitochondrial activities by injection of ooplasm from healthy donor [44]. It could avoid the chaotic mosaicism associated with low mitochondrial potential [43]. Liu et al. first reported the transfusion of active mitochondria in totally reconstructed zygotes with nuclei and cytosol from H_2_O_2_ treated as well as untreated zygotes that ultimately showed TUNEL staining at the similar rate as that of H_2_O_2_ treated controls, indicating that apoptotic potential could have transferred cytoplasmically [45]. This experiment also revealed that healthy cytoplasm could partly rescue pronuclei from OS.

#### Regulation of apoptosis

Apoptosis is a natural and normal feature in preimplantation development, both in vivo and in vitro, which is essential in the developing embryo for removal of deficient cells. Embryo development is controlled by counterbalance between pro-(i.e. Bax) and anti-apoptotic *(*i.e. Bcl-2) genes, as oogenesis, throughout the preimplantation period, and it precedes facing successive checkpoints [46]. Cytochrome C and apoptosis-inducing factor (AIF) are bifunctional proteins with oxidoreductase and apoptogenic functions, which frequently lead to the caspase cascade activation with changes in membrane asymmetry and disposal of phosphatidylserine (PS) [46]. Anti-apoptotic factors (i.e. Bcl-2) are inhibitors that naturally block the programmed cell death pathway [46]. When these factors mutated or incorrectly regulated, cancer or other complications may occur [46]. The impacts of OT on the balance between pro- and anti-apoptotic factors of recipient oocyte are regulated by some internal and external factors [46]. For instance, controlled ovarian stimulation can potentially result in inadequate ooplasmic maturation because of an uncoordinated apoptotic system [47–51]. Recent evidence suggested that consumption of oral antioxidants can non-specifically regulate the apoptosis process in oocytes which might be worthy before OT [52]. The growth hormone (GH) involved in ovarian regulation may reduce apoptosis during cumulus expansion and in-vitro culture (IVC), by altering the Bax: Bcl-2 ratio and increasing its DNA repair capacity [53–56]. Polluted air or smoking can induce toxicity mediated by a Bax pathway and alter the meiotic spindle of oocyte and sperm [57–60]. In oocyte and preimplantation human embryo, concentration of ROS and DNA fragmentation levels are comparatively more in fragmented than in non-fragmented forms because of inducing apoptosis [61–67]. This evidence suggests that most probably the mitochondrial component of ooplasm has the most significant role in mediating development and oxidative stress induced apoptotic cell death in zygote [29, 45, 51, 68–72]. It has been shown that because of some gene activations and productions in culture media, the level of apoptosis in this condition was higher than in in vivo condition [48, 73–76]. There is controversy surrounding supplementation of culture medium with amino acids and exogenous growth factors such as transforming growth factor-b (TGF-b), insulin-like growth factor I (IGF-I) or platelet-activating factor (PAF; 1-o-alkyl-2-acetyl-sn-glycero-3-phosphocholine) which are supposed to reduce sulphur-free amino acids to induce the apoptosis [74, 77–86]. Ceramide and sphingosine-1-phosphate (S1P), as a mediator and a suppressor respectively, are involved in oocyte apoptosis [87–89]. Mammalian oocytes and preimplantation embryos have many transcripts encoding cell death suppressor, inducer and caspase genes during developmental stages [49, 90]. In preimplantation human embryos, caspases are associated with developmental processes [91, 92]. It was suggested that the embryo development is regulated by an equivalence of pro- and anti- apoptotic gene expression with sequential checkpoints [48]. Mature oocytes with adequate anti-apoptotic mRNA have appropriate maturation and fertilization [48, 93]. Also, poor quality oocytes with increasing cell death inducers or decreasing its antagonists may have fragmentation or arrest [93]. When pronuclei are formed, expression of mRNA that codes for DNA repair enzymes may be observed as a marker of oocyte quality [94–99]. Transfer of ooplasm can also introduce unwanted menacing elements by elevating the level of apoptosis in the embryo; it seems that transmission of about 15% of ooplasm cannot adjust the death promoters and inhibitors [93]. Several studies suggested that OT might increase the capacity of DNA repair by transfer of the essential repair enzymes, like polymerase beta or the multifunctional DNA repair enzyme APEX [29, 100]. It is obvious that ooplasm donated from a healthy oocyte provides specific endogenous growth factors crucial for survival, mRNA and anti-apoptotic mRNA in women experiencing advanced reproductive age or poor embryo quality [18, 21, 101]. It seems that OT might increase the essential repair enzymes and apoptosis and the anti- or pro- apoptotic factors in ooplasm. Due to inadequate clarity in proper identification of the process of apoptosis and the anti- or pro- apoptotic factors in the preimplantation embryo, OT is applied on a complete empirical basis.

#### Genetic modifications

Though most of the reports describing genetic alterations of OT point out towards its beneficial impacts, still few reports discuss the concerns associated with OT. Reports emphasizing upon the positive impact of OT divulge its assistance in proper oocyte cell division and in reduction of probability of chromosomal abnormalities. Two major proteins closely linked to dynamics of microtubules in ooplasm are mitogen-associated protein kinase (MAPK) and M-phase promoting factor (MPF) [11, 12, 102–107]. MAPK as a serine/threonine-specific protein kinase is involved in directing cellular responses to the different stimuli and regulate cell functions [108]. MPF is the main regulator of germinal vesicle breakdown (GVBD) and chromosome condensation and mostly related with chromosomes and spindle structures (3, 4). Matured MII oocytes contain MPF and cytostatic factor (CSF) [3, 4]. CSF is an ooplasmic factor found in unfertilized eggs that causes metaphase arrest of cell cycles in the oocyte and zygote [109]. CSF appears in maturing oocyte ooplasm, but disappears during egg activation [109]. The effect of CSF appears to be primarily stabilization of MPF [109]. It has been proposed that the aged oocytes may have changes in ooplasmic capacity for spindle formation during meiosis [103–107, 110–112]. It was thought that the meiosis I and meiosis II spindles may be easily disturbed by the needling in OT and the disturbances can lead to the presence of multiple nuclei in a single oocyte and they are strongly correlated with embryo chromosomal abnormalities [113–115]. Gametes with these anomalies can result in conditions such as Down’s syndrome (47 chromosomes), or Turner syndrome (45 chromosomes). These abnormalities are frequently observed in manipulated preimplantation embryos and associated with diminished cleavage, poor quality, increased fragmentation and decreased implantation potential [113–115]. Although it has been proposed that OT can dynamically increase ooplasmic microtubules and cell cycle factors, it can form an atypical number of chromosomes or a structural abnormality in one or more chromosomes in embryos [20, 33, 107, 112]. In documenting the disadvantages of this technique in genetic alterations, the report of Liang et al. (2009) should be indicated which delineated the effect of OT on paternal genome functions [116]. By using C57BL/6 and DBA/2 mice strains, they have indicated that inter-strain OT significantly affects development of IVF/ICSI embryos as well as the paternal genome functions which could contribute to an altered phenotype after birth [116]. They showed that although OT can assist proper oocyte cell division and reduction of probability of chromosomal abnormalities by improving the ooplasmic capacity for spindle formation in poor oocytes, but meiosis I and meiosis II spindles may be easily disturbed by the needling and lead to embryo chromosomal abnormalities.

#### Epigenetic modifications

It was demonstrated that the parental genotype can influence the DNA methylation in genomic imprinting of the offspring mediated by S-adenosyl methionine (SAM) in CpG islands and regulate gene expression by creating hetero- and eu-chromatin [117–119]. In vitro culture conditions, inhibitors of SAM synthesis and deficiency in the endogenous methionine pool can also alter this genomic activation process and significantly affect embryo development and apoptosis [79, 80, 120]. Epigenetic programming plays multiple roles in post fertilization in embryo. Recent studies demonstrated that blastomere fragmentation in embryos is controlled by multiple genetic loci which were affected by genomic imprinting or different mitochondrial origin [121–123]. However, undoubtedly in OT, the foreign ooplasm and specific manipulations can have effect on epigenetic markers of oocyte and alter gene expression patterns and phenotype at later stages of development, and these effects immediately adverse early embryo development through blastomere fragmentation and apoptosis [117, 124–128]. Although the use of epigenetic analysis is rapidly expanded as a marker to reduce abnormal embryo morphology and reproductive ART outcomes, the analysis of the alteration of epigenetic markers is essential.

#### Role of oocyte contents in the embryo development

Oocyte is an electrogenic cell with specific developmental blockage during the meiotic division stages [129]. The quality of the ooplasm depends on maternal transcripts, proteins and ions accumulated during oocyte growth and maturation, until the maternal-to-zygotic transition (MZT) happens during the 4–8-cell stage and will establish the future of the embryo [130]. The evidence showed that potassium (K^+^) and calcium (Ca^2+^) are two important ions involved in different maturation stages of the oocyte [131, 132]. Wonky oocytes undergo several changes including altered intracellular K^+^ and Ca^2+^ dynamics, premature cortical granule loss and alteration in cell cycle factors and are more sensitive to undergo abnormal fertilization and arrest during early embryonic development [103, 110, 133, 134]. Studies on GVBD stage oocytes suggested that immature oocytes have a high K^+^ current which is lost during maturation [129]. Depolarization of granulosa cells is an essential event of oocyte maturation [129]. Because of cumulus expansion and the breaking of heterologous gap junction communications, this coupling decreases at the end of maturation [129]. In association with rapid modifications of the intracellular environment such as changes in free Ca^2+^ concentration, resumption of meiotic arrest due to hormonal signals and sperm entry, a wide range of changes occurred on the oocyte plasma membrane [129]. Studies showed that unfertilized oocyte contains unstable CSF, which is highly sensitive to Ca^2+^ [109]. As mentioned, CSF is responsible for the meiosis arrest in unfertilized oocytes, and its inactivation by a surge of Ca^2+^ during fertilization releases the oocyte from this arrest [109]. Methionine and cysteine can affect glutathione levels which prevent the negative effects of ROS and apoptosis in embryos [29, 80, 118, 135, 136]. Recent evidence suggested that the polarized protein like leptin and signal transducer and activator of transcription 3 (STAT3) and maybe beta-actin and IL-1 mRNAs can display polarized distribution in oocytes [137–139]. The pattern of polarization is inherited differentially between blastomeres in the cleavage-stage embryo and between the inner cell mass and trophoblast of the blastocyst [137–139]. However, this polarization phenomenon exists during early human development for transforming growth factor b2 (TGFb2), vascular endothelial growth factor (VEGF), apoptosis-associated proteins (Bcl-x and Bax), growth factor receptors like c-kit receptor and epidermal growth factor receptor (EGF-R), and loss of non-polarized proteins (i.e. E-cadherin) has no developmental consequences [29, 140]. It seems that the place of removing ooplasm from the donor and the place of injecting to recipient oocyte may be important because the injection of foreign ooplasm into a polarized domain in the recipient can be hazardous for subsequent embryo development if it alters regulatory protein polarization in the early preimplantation embryos and might have a clinical significance in OT. In 2016, Gonzalez-Grajales et al. have driven several doubts away reaching a convincing concept that OT and interspecies somatic cell nuclear transfer (iSCNT) did not have any detrimental effect on reconstructed embryo, as they did not observe any significant alterations in ATP content, expression of nuclear respiratory factor 2 (NRF2), mitochondrial transcription factor A (TFAM) and mitochondrial subunit-2 of cytochrome C oxidase (mtCOX2) in 8–16 cell bison embryos that emerged from the transformed cell [141]. They showed that OT may provide the suitable amounts of necessary ooplasmic contents which are responsible for the division in fertilized oocytes. And because of the oocyte polarization, the place of removing ooplasm from the donor and the injecting to recipient oocyte is important for subsequent embryo development.

#### Survival of required mRNA

Maternal mRNA translation is responsible for the early cleavages, zygote controlling and activation of embryonic genome [142]. The two important mechanisms involved in the recruitment of maternal mRNA are the stability of the message and its translation potential. Oocyte maturation is linked to a specific group of mRNA transcripts which continues after fertilization with different messages for degradation [143]. Rapidly or delayed degradation of oocyte mRNA is regulated via the length of its poly (A) tail which is affected by cytoplasmic polyadenylation element binding protein (CPEB), cleavage and polyadenylation specific factor (CPSF) and poly(A) polymerase (PAP) [144, 145]. Specific kinetic changes in polyadenylation contribute to gene expression in embryos, and these changes influence further development [146–148]. Inadequate ooplasmic maturation is linked to poor regulation of the mRNA polyadenylation process, and this leads to altered developmental competence [146–148]. For instance, the mRNA translation of Oct-4, connexin-32 and connexin-34, essential for early differentiation, totipotency and blastocyst formation, are protected via PAP [29]. Injection of good quality ooplasm might be beneficial by assisting the process of polyadenylation via injection of PAP, CPEB, CPSF and some polyadenylated mRNA [29]. On the other hand, some of the polyadenylated mRNAs gradually reduce their poly A tail (connexin-43 and Oct-4), and injection of exogenous factors could disturb the developmental process of embryonic cell cycle [29]. In addition to positive effects, it is believed that extra foreign ooplasm might interfere during the MZT cycle. Therefore, on the basis of mRNA status in recipient and donor ooplasm, OT is of questionable value.

## Conclusion

It was observed that although an OT technique can practically establish normal growth and return viability to the embryos, but the appropriate practical OT techniques in human oocytes, and evaluating its positive and negative aspects in mitochondrial functions, regulation of apoptosis, genetic and epigenetic modifications, oocyte contents and survival of required mRNA are necessary. If an unhealthy oocyte with intact nuclear entity, lacking some potent cytoplasmic factors can participate in reproduction, aggrandising the oocyte by the required ooplasmic factors from another donor oocyte, life can be bestowed to innumerable infertile wombs. Thus, OT with efficient technicality may be accepted as a boon to the reproductive arena. Although the intervention of mtDNA of the donor in the transformed oocyte and eventually into the zygote and mtDNA heteroplasmy in the formed fetus may create concerns suggesting an ethical burden in the concept of ‘three genetic parents’ of the fetus immerging from the transformed oocyte, it is also evident from this review itself that no significant impact of mtDNA from the donor on the formed fetus has so far been reported with results strengthening the reliability of OT emphasising mitochondrial homoplasmy with almost no genetic intervention of donor oocyte. Moreover, as mentioned, OT can dynamically increase ooplasmic microtubules and cell cycle factors and alter the genetic and epigenetic factors. It was shown that because spontaneous mtDNA heteroplasmy occurs in normal individuals, there is no reason to worry about its detection in recipient oocytes so injection of ooplasm from healthy donor could potentially restore mitochondrial activity. In addition to these factors, the place of removing ooplasm from the donor and the place of injecting to recipient oocyte may affect the subsequent embryo development. The lack of an objective biomarker in recipient oocyte is one of the concerns to identify dysfunctional oocytes without testing their developmental ability. These worries also raise the question of how suitable oocytes for donating ooplasm should be identified or defined. It is obvious that criteria that will allow this evaluation are not yet available for clinical usage and will only be elucidated through fundamental basic research. Thus, removing the few concerns surrounding the implication of OT and with more technical improvement and standardization, OT may lead to a successful contribution in changing unhealthy into healthy oocytes enabling assisted reproduction to progress further in making new lives.

## Abbreviations

AIF: Apoptosis-inducing factor
Ca^2+^: Calcium
CPEB: Cytoplasmic polyadenylation element binding protein
CPSF: Cleavage and polyadenylation specific factor
CSF: Cytostatic factor
GH: Growth hormone
GV: Germinal vesicle
GVBD: Germinal vesicle breakdown
ICSI: Intracytoplasmic sperm injection
IGF-I: Insulin-like growth factor I
iSCNT: Interspecies somatic cell nuclear transfer
IVC: In-vitro culture
K +: Potassium
MAPK: Mitogen-associated protein kinase
MII: Second meiotic metaphase.
MPF: M-phase promoting factor.
mRNAs: Messenger RNAs.
mtCOX2: Mitochondrial subunit-2 of cytochrome C oxidase.
mtDNA: Mitochondrial DNA.
MZT: Maternal-to-zygotic transition.
nDNA: Nuclear DNA.
NRF2: Nuclear respiratory factor 2.
OT: Ooplasmic transfer.
OXPHOS: Oxidative phosphorylation.
PAF: Platelet-activating factor.
ROS: Reactive oxygen spaces.
S1P: Ceramide and sphingosine-1-phosphate.
SAM: S-adenosyl methionine.
STAT3: Signal transducer and activator of transcription 3.
TFAM: Mitochondrial transcription factor A.
TGF-b: Transforming growth factor-b.
TGFb2: Transforming growth factor b2.
VEGF: Vascular endothelial growth factor.
